# Broad-Spectrum Virucidal Activity of Polymer Cryogel-Loaded Formic Acid Against a Panel of Naked and Enveloped Viruses

**DOI:** 10.3390/ijms27115145

**Published:** 2026-06-05

**Authors:** Desislava Budurova, Petar D. Petrov, Filip Ublekov, Miroslav Metodiev, Lora Simeonova

**Affiliations:** 1 Institute of Polymers, Bulgarian Academy of Sciences, “Acad. G. Bonchev” Street, Bl.103A, 1113 Sofia, Bulgaria; dbudurova@gmail.com (D.B.); ppetrov@polymer.bas.bg (P.D.P.); ublekov.philip@gmail.com (F.U.); 2 Stephan Angeloff Institute of Microbiology, Bulgarian Academy of Sciences, 26 G. Bonchev Street, 1113 Sofia, Bulgaria; metodiev.1996@abv.bg

**Keywords:** formic acid, PNIPAm cryogel, controlled release, virucidal, disinfection

## Abstract

Viruses cause a great number of infectious diseases with medical, veterinary, agricultural, social and economic impact. Their unique mechanisms to spread, overcome and resist the existing countermeasures require innovative and smart antiviral strategies such as the effective disinfection of enclosed environments with ensured broad-spectrum efficacy and minimized risks associated with handling liquid biocides. Formic acid (FA) is a well-established natural acaricide used in beehives with an antiviral potential; however, its application in a liquid form is hindered by severe corrosiveness and rapid, uncontrolled evaporation. This study describes a novel formulation of FA, using a cryogel carrier for achieving a vapor-phase inactivation of viruses, thus eliminating the need for direct contact between the disinfectant and the pathogen. Firstly, a poly(N-isopropylacrylamide) (PNIPAm) cryogel was synthesized by a procedure involving cryogenic treatment, photochemical crosslinking, and freeze-drying, and then the cryogel was swollen with 65% FA or _dd_H_2_O as a control. After an exposure of a panel of animal and human viruses to FA, evaporated by the polymer carrier for time intervals between 15 min and 12 h, they were neutralized completely as follows: Poliovirus (PV) as a surrogate for major bee viral pathogens for 60 min by 5.1 ∆lg; Feline calicivirus (FCV) for 60 min by 5.3 ∆lg; Adenovirus 5 (AdV5) for 12 h by 4.0 ∆lg; and Influenza virus A (IAV) for 15 min by 5.1 ∆lg. Results were recorded after titration, 48–72 h incubation, cytopathic effect estimation and NR uptake assay. Our results suggest that 65% FA, when delivered via the PNIPAm cryogel matrix, acts as a powerful agent for fumigation-like disinfection. This “dry” delivery strategy offers significant practical advantages: it eliminates the need for open liquid containers, prevents spill-related hazards, and provides an alternative for controlled, long-term release of active vapors.

## 1. Introduction

Formic acid (FA), also known as methanoic acid, is the simplest carboxylic acid found in plants, insects, and microbial systems. Its beneficial physicochemical properties such as relatively strong acidity among organic acids, high polarity, and ability to penetrate biological membranes lays under its broad antimicrobial activity and diverse applications in agriculture, food preservation, and veterinary medicine. In recent years, increasing emphasis on sustainable and residue-free control strategies has raised scientific interest in FA as a natural agent with significant biocidic potential. A major driver of this interest is the global impact of the parasitic mite *Varroa destructor*, a key biotic stressor implicated in honeybee (*Apis mellifera*) colony losses. This ectoparasite feeds on host tissues and acts as a vector for multiple viral pathogens, ultimately leading to colony collapse if left unmanaged [1]. Conventional synthetic acaricides have been widely used to control Varroa, but their long-term effectiveness is increasingly compromised by resistance and residue concerns. Extensive studies demonstrate that FA exhibits high acaricidal efficacy against V. destructor, with mortality rates exceeding 88.7–90%, depending on conditions. Among organic aids used in beekeeping, FA is unique as it acts simultaneously as an acaricidal, fungicidal and a virucidal agent [2]. Its volatility enables penetration into capped brood cells, targeting reproducing mites [3,4], and the rapid degradation also reduces contamination risks in hive products [5]. Genath et al. conducted a proteomic analysis showing that FA treatment disrupts Varroa destructor mites by alterations in proteins related to detoxification, oxidative stress, and metabolic functions, indicating a multifaceted toxic effect. However, the full molecular mechanisms remain incompletely understood and require further study [6]. Despite the numerous advantages of FA against the Varroa mite, the potential hazardous effects on honeybee colonies and development of resistance under the selective pressure of rapid and uncontrolled release of high dosage of the compound should be considered. Studies investigating factors such as adjuvants, application timing, dosage rates, and formulation stability can improve the practicality and effectiveness of organic acaricide treatments [7].

Beyond its direct virulence on bees, Varroa transmits a number of viral pathogens, which infect and damage apicultures. So far, about 18 different viruses have been isolated from honeybees, and many of them, such as Kashmir bee virus (KBV), Sacbrood virus (SBV), Acute bee paralysis virus (ABPV), Israeli acute paralysis virus (IAPV), and Deformed wing virus (DWV), are vectored by Varroa mites not only mechanically but with an active replication in vector, too [8]. Viruses could attack at different developing stages and castes of the insects, including eggs, larvae, pupae, adult worker bees, adult drones, and queens of the colonies. Best known is the DWV infection causing the typical symptoms of crippled wings and shortened abdomen in heavily infested honeybee colonies [9,10].

All honeybee viruses have positive-sense single-stranded RNA genome, icosahedral, pseudo T = 3 structure symmetry. They lack lipid-containing envelopes and range in size from 17 to 30 nm. Most of them belong to the picorna-like virus superfamily. The outer shell of the capsid is composed of 60 capsomers, each consisting of a single molecule of three subunits: VP1, VP2, and VP3. There is a smaller fourth protein VP4 that is present in the virions of some viruses such as BQCV and ABPV. The capsid proteins play important roles in the protection of viral RNA from activities of RNases and irregular environments and in the determination of viral host specificity and tissue tropism [11]. The transmission takes place horizontally by airborne, foodborne, fecal–oral, venereal or Varroa-borne routes, as well as vertically. The honeybee colony acts as a single unit to share labor, specialize in tasks, and coordinate efforts. The homogeneous genetic structure, close physical contact, and extensive social interactions among individuals and infestations make bees especially vulnerable to the infection and transmission of pathogens [12].

Virucidal agents are chemical compounds capable of inactivating viruses outside the host cell or organism. The well-established and most commonly applied in practice standard acaricides—65% FA and other natural acids such peracetic acid, involved in disinfectants—have demonstrated promising virus-destructing activity against several naked and enveloped viral models of human and animal diseases, with some serving as surrogates of major bee pathogens [13,14]. Antiviral effects of FA against porcine epidemic diarrhea virus (PEDV), with reductions in viral replication, were observed in vitro [15]. Disinfectant formulations containing FA were also efficacious against African swine fever virus (ASFV), achieving >4 log10 reductions under practical conditions [16]. Additional studies confirm its potential against Enterovirus, Newcastle disease (NDV), porcine herpes virus, avian influenza virus, bovine viral diarrhea virus, equine rhinitis A virus and porcine parvovirus in surface and animal by-products disinfection [17,18].

Viral disease outbreaks or chronic infections in the beekeeping industry can seriously affect its profitability, but it is not the only field it poses a challenge to. In fact, the effective prevention, control and management of viral diseases in agriculture, veterinary and human medicine necessitates smart and adaptive strategies to overcome serious barriers such as toxicity, carcinogenicity, ecosafety, resistance emergence and virulence increase among pathogens by dosage forms of both synthetic and natural compounds used [19,20,21,22,23].

Currently, polymeric carriers play a key role in modern biomedical and nanomedicine applications [24]. Various polymer delivery systems, such as hydrogels and nanoparticles, have been successfully applied for the controlled delivery of antimicrobial or bioactive agents [25,26,27]. Polymeric cryogels are of particular interest as carriers for water-soluble agents due to their unique structure. They are super-macroporous materials obtained by freezing and thawing aqueous solutions containing monomeric or polymeric precursors [28]. The hydrophilic nature and sponge-like structure of cryogels allow for the immobilization of various biomolecules and active agents, as well as their controlled release [29].

In the present work, we propose an original approach to test virucidal activity of organic acids. A novel macroporous PNIPAm cryogel is used as a model system and an innovative carrier for the controlled release of formic acid. Through the developed original gas-phase testing methodology, based on the separation of the gel matrix from direct contact with viral particles, we demonstrate that the gaseous state is sufficient to achieve high virucidal effects.

## 2. Results

### 2.1. Formic Acid Cumulative Release Kinetics

**Figure 1:**
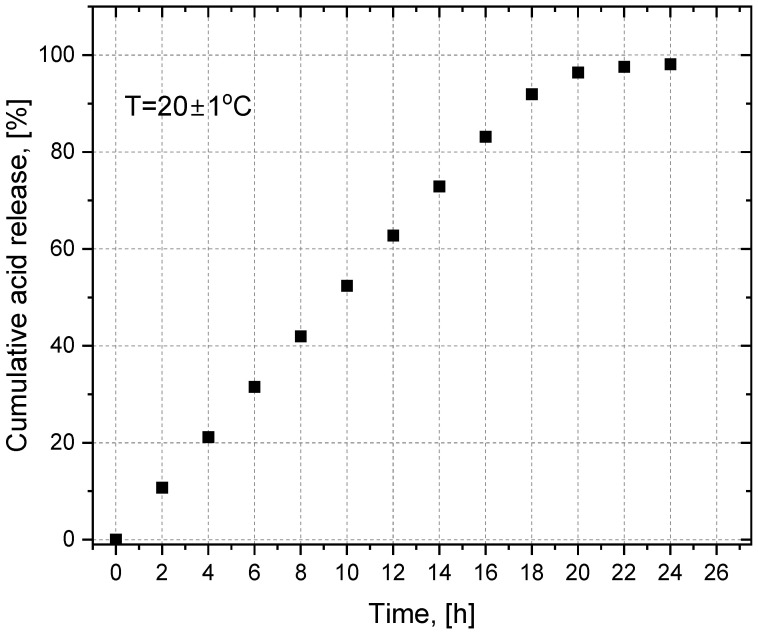
Cumulative release kinetics of formic acid (FA) from the PNIPAm cryogel matrix.

Based on continuous gravimetric measurements conducted at 20 ± 1 °C within a scaled, closed volume, the experimental data (Figure 1) reveals a well-controlled release profile of FA from the PNIPAm cryogel matrix. The cumulative release followed a highly predictable pattern, reaching near-complete exhaustion (~98.1%) by the 24 h mark. Notably, no initial burst effect was observed, indicating that the super-macroporous gel network effectively anchors the acid molecules, ensuring a gradual and safe transition to the vapor phase.

### 2.2. Cytotoxicity and Antiviral Activity

Cytotoxicity and antiviral effect were tested with liquid formic acid at serial dilutions. Strong cytotoxic effects were observed up to 0.1% FA concentration when tested in both epithelial MDCK and HEp2 cells with no survival, after they were incubated in presence of the substance for 48 h at 37 °C in CO_2_ thermostat. CC_50_ values of FA calculated were similar in both epithelial lines, as in MDCK cells they were 0.075% and in HEp2 0.065%. Microscopy visualized mild changes in cellular morphology at 0.05% concentration, but they did not affect cell viability according to NR uptake assay, and it was determined as MTC. Further evaluation of 0.05% FA concentration revealed no inhibitory effect on the 48–72 h replication of the viruses studied, and cytopathic effect (CPE) was comparable to that in the untreated viral control.

### 2.3. Virucidal Activity (Effect on Extracellular Virions)

**Figure 2:**
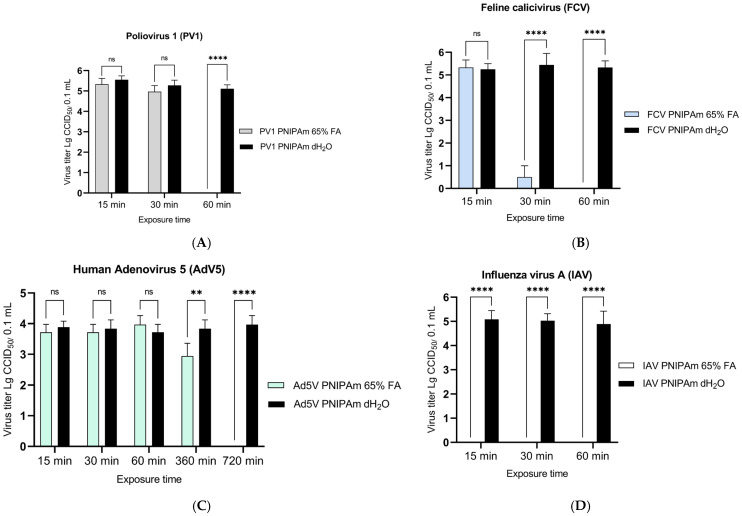
(**A**–**D**). Virucidal activity of 65% FA loaded in PNIPAm cryogel against naked (**A**–**C**) and enveloped (**D**) viruses. Error bars—standard deviation (SD) of the mean titers calculated from three replicates. **∆lg**—difference beween PNIPAm loaded with 65% FA and _d_H_2_O-loaded control. Statistical analysis: Two-way ANOVA with Šídák’s multiple comparisons post-test; ns—not significant; ** *p* < 0.01; **** *p* < 0.0001.

In the next panel of experiments, we used the 65% FA as a standard accepted and applied for disinfection and anti-acar agent in apiculture triggered by the hypothesis and literature data of possible virucidal capacity of the compound. Because of the high cytotoxicity, corrosive effects and rapid evaporation, we selected PNIPAm cryogel as a matrix, and loaded it with FA for slow release without drastic environmental pressure in the internal ecosystem. We tested poliovirus 1 (PV1) and feline calicivirus (FCV) as +RNA non-enveloped viruses as structurally similar to the major bee viral pathogens, transmitted by Varroa. Adenovirus 5 (AdV5)—DNA naked virus was included as the most stable virus known, which usually needs prolonged and highly intensive treatment compared to others. Influenza virus was tested to complete the panel as an enveloped representative as a primary idea and proof of concept that is more sensitive to acidic chemicals and deactivated more rapidly. As shown in Table 1 and Figure 2A–D, IAV was destructed completely for 15 min (5.1 ∆lg), and FCV and PV1 needed 60 min (5.3 ∆lg) (5.1 ∆lg), respectively, to lose their infectivity. As suggested, the most durable model was AdV5, requiring 12 h (highest concentration of FA vapor) to be completely inactivated (Table 1, Figure 2A–D).

**Figure 3:**
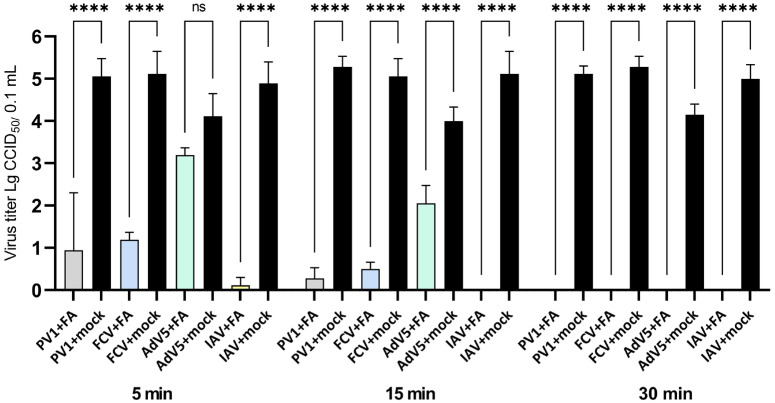
Effect of 65% liquid formic acid (FA) on extracellular viral particles mixed at a ratio of 1:1 with various naked and enveloped RNA and DNA virus suspensions. **∆Lgs** are calculated as the difference with DMEM viral control (mock). Statistical Analysis: Values are expressed as titers (CCID_50_/0.1 mL) determined for each time point. Mean of three independent experiments ±SD. Two-way ANOVA with Šídák’s multiple comparisons test **** *p* < 0.0001; ns—not significant.

To verify its direct virucidal properties, we also studied liquid formic acid at its maximum concentration of 65% in a suspension test, where it was diluted twice to the final concentration of 32.5% when mixed with an equivalent amount of viral suspension (Table 2). All four viruses were completely deactivated for their infective abilities for up to 30 min contact time, with IAV blocked with more than 4.0 ∆Lgs (4 lgs is considered as standard in virucides testing) at 5 min and with 5.11 ∆Lgs after 15 min. PV1 and FCV were significantly reduced again, with over 4.0 ∆Lgs after 15 min and fully neutralized for 30 min of contact with FA. AdV5 needed 30 min for maximum antiviral effect (Figure 3). For all the viral models evaluated, statistically significant, no sign of infectious viral particles was observed at the 30th min of incubation in the suspension test performed and subsequent titration by end-point dilution method.

## 3. Discussion

PNIPAm hydrogels are widely studied as carriers of active substances for drug delivery, tissue engineering, wound healing, etc. [30]. In particular, chemical crosslinking of PNIPAm with appropriate crosslinking agents provides materials with good mechanical stability and strength, as well as high biocompatibility, which is essential for biomedical applications. In addition to water-based gels, the PNIPAm network can also swell in organic solvents (organogels), as the process mainly depends on the ability of the solvent to form hydrogen bonds with the pendant groups of the polymer chain [31]. In the case of FA, the possible mechanism involves donor/acceptor interactions between the acidic −OH group (hydrogen bond donor) and −C=O group (acceptor), binding with the −N-H (donor) and −C=O of PNIPAm, respectively.

In the present study, PNIPAm cryogel (super-macroporous hydrogel) was chosen as a model carrier of 65% FA, not only because of the polymer’s affinity for FA, but also because of its good mechanical strength and structural stability. Furthermore, the specific structure of the cryogel, consisting of huge, interconnected pores and thin polymer walls [32], allowed us to increase the swelling capacity of the material more than twofold compared to the conventional PNIPAm hydrogel [32].

Kinetic analysis of the release profile demonstrated a remarkable adherence to strict zero-order kinetics extending over an initial 16 h period (up to ~83% cumulative release). In this prolonged steady-state phase, the linear regression yielded a high coefficient of determination (R2 > 0.99) and a constant release rate (K0) of 5.23% h^−1^. To further elucidate the release mechanism, the fractional release data was fitted to the Korsmeyer–Peppas mathematical model (Mt/M∞ = Ktn). The calculated diffusional exponent was n ~1.0, which is the theoretical optimum for a Case II transport mechanism. This confirms that the FA evaporation is entirely decoupled from simple concentration-gradient diffusion and is exclusively governed by the macromolecular relaxation and structural dynamics of the super-macroporous PNIPAm chains. Only after the 16th hour did the release rate gradually decelerate into a first-order phase. This prolonged, 16 h zero-order delivery confirms that the cryogel acts as a highly reliable continuous reservoir [33,34,35,36].

Antimicrobial properties of natural organics against viruses, bacteria, fungi, and molds have been extensively studied [37]. Enveloped viruses in general are much more acid-sensitive than those without lipid supercapsid. They are more easily destructed by low-pH treatment, but virucidal efficacy against non-enveloped viruses, bacteriophages included, has also been well documented [38,39]. The mechanism of inactivation is majorly related to the acid’s denaturating action on the viral structural lipids, glycolipids, proteins and glycoproteins, leading to conformational and functional disability. Salts present in the solution might also play a role by viral particle cluster formation and subsequent loss of infective properties [40]. Peracetic, citric, salicylic, pyroglutamic and formic acid have shown virucidal activities against a broad-range of non-enveloped viruses and pathogens, with effects being dependent on the virus nature, temperature, acidity and time of contact. In particular, formic acid has shown efficacies against vaccinia virus (VV), bovine viral diarrhea virus (BVDV), rhinovirus, African swine fever virus (ASFV), enterovirus and others, and is considered as a potential antimicrobial agent in animal feed, and industrial and household cleaning [9,16,41,42]. Our data showed that IAV was inactivated completely with ∆lg = 5.1 for 15 min of contact with delayed-release 65% FA, followed by FCV and PV1, which needed 60 min for full neutralization with ∆lg = 5.3 and ∆lg = 5.1, respectively. The naked dsDNA Ad5V appeared to be the most acid-resistant pathogen, with 12 h incubation needed to achieve the maximum effect of ∆lg 4.0. The results are consistent with the literature data on virus structure–contact time.

## 4. Materials and Methods

### 4.1. Materials

N-isopropylacrylamide (NIPAm, 97%), poly(ethylene glycol) diacrylate (PEGDA, average molar mass = 575 g/mol), hydrogen peroxide (H_2_O_2_, 30 vol.% water solution) and formic acid (98–100%) were purchased from Sigma-Aldrich (St. Louis, MO, USA) and used without purification.

### 4.2. Syntheses of PNIPAm Cryogel

Monomer N-isopropylacrylamide (1 g), crosslinking agent PEGDA (0.1 g; 10 mass % relative to monomer) and initiator H_2_O_2_ (0.167 mL; 5 mass % relative to monomer) were dissolved in 10 mL of deionized water at room temperature under stirring to obtain a homogeneous aqueous solution. The solution was divided into 10 portions of 1 mL each, poured into 10 Teflon dishes (20 mm diameter) and stored in a freezer for 2 h at a temperature of −20 °C. Next, the frozen samples were irradiated with a full-spectrum UV–vis light (Dymax 5000-EC’’ UV curing equipment (Dymax Corporation, Torrington, CT, USA), with 400 W metal halide flood lamp) for 5 min at an irradiation dose rate of 5.7 J/cm^2^ min (input power of 93 mW/cm^2^). Finally, the PNIPAm cryogels were freeze-dried.

### 4.3. Preparation of Formic Acid-Loaded PNIPAm Cryogels

Each freeze-dried gel sample was accurately weighed and immersed in an excess volume of 65 wt.% aqueous FA solution in a hermetically sealed glass vessel. To achieve a uniform distribution of the acid throughout the polymer network, the samples were incubated at room temperature for 24 h. The amount of immobilized acid was determined gravimetrically. The swelling capacity of the PNIPAm cryogel in 65 wt.% aqueous FA solution was determined to be 920% ± 60%, based on the average of five independent measurements. The swelling ratio was calculated according to the following equation:

Swelling ratio (%) = [(Ws − Wd)/Wd] × 100%

where Ws and Wd are the mass of swollen and dried cryogels, respectively.

The resulting formulations were stored in airtight containers for further release-rate analysis and testing.

### 4.4. Determination of Formic Acid Cumulative Release Profile

The cumulative release profile of FA was determined gravimetrically within a scaled, closed model hive. The release kinetics were measured at a temperature of 20 °C ± 1 °C, with weight change measurements recorded at 1 min intervals. All data were collected and processed via automated software Precisa^®^Channelizer, Version 1.0 (Precisa Gravimetrics AG, Dietikon, Switzerland).

### 4.5. Cells

Human laryngeal squamous cell carcinoma cells (HEp2) and Crandell–Rees Feline Kidney (CRFK) cells were obtained from National Bank for Industrial Microorganisms and Cell Cultures, Sofia, Bulgaria. Madin–Darby canine kidney (MDCK, ATCC-CCL-34™) cells were purchased from American Type Culture Collection, Manassas, VA, USA. The three cell lines were grown in DMEM containing 10% FBS and supplemented with 10 mM HEPES buffer and antibiotics (100 IU/mL penicillin, 100 μg/mL streptomycin, 25 μg/mL amphotericin B) (Sigma-Aldrich, St. Louis, MO, USA). All the cells were incubated in a humidified atmosphere of 5% CO_2_ at 37 °C using a Thermo Scientific Forma™ 310 CO_2_ thermostat (Thermo Fisher Scientific, Waltham, MA, USA).

### 4.6. Viruses

#### 4.6.1. Non-Enveloped/Naked Viruses

Poliovirus 1(PV1), LSc-2ab strain, was selected and served as a surrogate model for bee viral pathogens due to their high resemblance of structural VP capsid proteins. PV1 together with Human adenovirus type 5 (HAdV-5) were initially obtained by the District Center for Hygiene and Epidemiology, Plovdiv, Bulgaria and kindly provided by Dr. I. Nikolova, Institute of Microbiology, BAS for the analysis. Feline calicivirus (FCV) originated from the collection of Stephan Angeloff Institute of Microbiology, Bulgarian Academy of Sciences, Sofia, Bulgaria.

HCV was propagated in CRFK with an infectious virus titer (Tlg) of 10^5.5^ CCID_50_/0.1 mL. PV1 was grown in HEp2 cells (T = 10^7.5^ CCID_50_/0.1 mL), as well as HAdV5 (T = 10^4.0^ CCID_50_/0.1 mL).

#### 4.6.2. Enveloped Viruses

MDCK-cultivated seasonal influenza A virus (IAV) (strain A/Panama/07/99 (H_3_N_2_) from the National Center for Infectious and Parasitic Diseases—NCIPD, Sofia, Bulgaria) was used at a titer of T = 10^5.25^ CCID_50_/0.1 mL.

Three of the viral infections in permissible cells were performed with undiluted cell-derived stock viral suspension (FCV; AdV5and IAV), but PV1 was used at 10^−2^ dilution in maintenance Dulbecco’s modified Eagle medium.

### 4.7. Cytotoxicity and Antiviral Assay

Cytotoxicity of formic acid in HEp2 and MDCK cells and antiviral effect against the five viruses was evaluated at the 48th–72nd hour. Visual microscopic observation of morphological alternations and virus-induced CPE (cytopathic effect) was followed by cell viability assessment after treatment with varying concentrations of the compound with subsequent neutral red (NR) dye uptake assay as described previously [19]. The optical density (OD) of each well was measured at 540 nm using a microplate reader (Biotek Organon, West Chester, PA, USA), and CC_50_ (50% cytotoxic concentration) was estimated as the concentration causing 50% cell damage as compared to the healthy cell control. Maximum tolerated concentration (MTC) was also determined.

### 4.8. General Procedure for Virucidal Activity Testing

The tested PNIPAm polymer loaded with 65% FA (Sigma-Aldrich^®^, St. Louis, MO, USA) or distilled water as a control at an amount of 75 mg was placed in a 15.8 mL volume available for the fumigation in the experimental well, where 1 mL of undiluted viral suspension was exposed to the evaporating compound at 20 °C. The scaling was based on a conventional field dose of 200 g of 65% formic acid, a treatment regimen widely utilized in apicultural practice for the effective control of Varroa mites. The exposure lasted for 15, 30, 60, 120, 360 and 720 min, as at each time point sampling was performed. In a separate set of experiments, the activity of 65% liquid FA (32.5% final concentration) in a suspension test was determined when combined in a 1:1 ratio with 1 mL of the same viral agents. The samples were stored at room temperature for 5, 15, and 30 min intervals. The residual infectious virus content in each sample was determined by the end-point dilution method in the permissive cell cultures for each virus and ∆lgs compared to the infected and mock-treated controls.

### 4.9. Determination of pH

For each time interval of exposure to formic acid vapors emitted from the PNIPAm cryogel, as well as in the control samples with _d_H_2_O-loaded polymer, the acidity in viral suspension was measured using MilliporeSigma™ MQuant™ pH Test indicator strips (Thermo Fisher Scientific, Waltham, MA, USA) with differentiated color gradations (0.5 units) for a more precise evaluation.

### 4.10. Statistical Analysis

Data from the spectrophotometric readings were recorded using Gen5^®^ v3.16 (BioTek Instruments, Winooski, VT, USA) and further processed by Excel^®^ 2016 Microsoft (Microsoft Corporation, Redmond, WA, USA). The values of CC_50_ were calculated using OriginPro 9.0^®^ (OriginLab Corporation, Northampton, MA, USA) and two-way ANOVA, and Šídák’s multiple comparisons post-test was applied for comparison of treated groups (GraphPad 9.0^®^ Software, San Diego, CA, USA). The values were presented as means ± SD from three independent experiments.

## 5. Conclusions

When discussing the practical application of high-concentration chemicals as biocides, even from natural origin, crucial pitfalls to overcome are the cellular and environmental toxicity, dose-dependent pressure on microorganisms leading to resistance, and corrosiveness. The release of the bioactive component under controlled and step-by-step conditions in an appropriate system is a reasonable strategy to avoid undesired contaminations and infections in medicine, agriculture and industry. Concerning volatile organic acids such as formic acid (FA), gel matrices provide a sustained evaporation profile as an alternative to the uncontrolled liquid–gas “burst” transition. This ensures that the fumigant remains within the optimal efficacy range for an extended period, while significantly increasing operational safety and application flexibility.

Our study demonstrates that fumigation from macroporous PNIPAm cryogel provides a stable, predictable, and safer delivery mechanism and works as an innovative and reliable method for studying contactless action of the vapor phase, emitted from the gel matrix against the selected viral models. By avoiding direct physical contact between fluids, we found a small-scale model verifying formic acid as a compound with both acaricide and virucide capacity, which might be used for development of approaches for prevention of beehives not only against Varroa but against major viral pathogens as well. Further progress in optimized delayed-release systems of dual-action substances may offer a major advantage for improving beehive health and reducing environmental and economic losses, establishing a framework for next-generation pathogen control.

## Figures and Tables

**Table 1:** Virucidal activity of PNIPAm cryogel-loaded 65% formic acid (FA) against a panel of naked and enveloped RNA and DNA viruses and pH determination.

Treatment	Viral Titers Lg CCID_50_/0.1 mL ± SD and Δlg *														
Viral Model ^#^	15 min	∆Lg	pH	30 min	∆Lg	pH	60 min	∆Lg	pH	360 min	∆Lg	pH	720 min	∆Lg	pH
PNIPAm + 65% FA + **PV1**	5.3 ± 0.3	0.2	5.0	5.0 ± 0.3	0.3	4.0	0.0	**5.1**	3.0	nd	-	nd	nd	-	nd
PNIPAm + _d_H_2_O + **PV1**	5.5 ± 0.2	-	7.0	5.3 ± 0.2	-	7.0	5.1 ± 0.2	**-**	7.2	nd	-	nd	nd	-	nd
PNIPAm + 65% FA + **FCV**	5.3 ± 0.3	0.0	4.8	0.5 ± 0.5	4.9	3.9	0.0	**5.3**	3.0	nd	-	nd	nd	-	nd
PNIPAM + _d_H_2_O + **FCV**	5.3 ± 0.3	-	7.0	5.4 ± 0.5	-	7.0	5.3 ± 0.3	**-**	7.0	nd	-	nd	nd	-	nd
PNIPAm + 65% FA + **AdV5**	3.7 ± 0.3	0.0	4.5	3.7 ± 0.3	0.1	4.2	4.0 ± 0.3	**−0.3**	3.2	2.9 ± 0.4	**0.9**	2.3	0.0	**4.0**	1.5
PNIPAm + _d_H_2_O + **AdV5**	3.9 ± 0.2	-	7.0	3.8 ± 0.3	-	7.2	3.7 ± 0.3	**-**	7.1	3.8 ± 0.3	-	7.0	4.0 ± 0.3	-	6.9
PNIPAm + 65% FA + **IAV**	0.0 ± 0.0	5.1	4.5	0.0	5.0	4.0	0.0	**4.9**	2.9.	nd	-	nd	nd	-	nd
PNIPAm + _d_H_2_O + **IAV**	5.1 ± 0.4	-	7.2	5.0 ± 0.3	-	7.1	4.9 ± 0.5	**-**	7.0	nd	-	nd	nd	-	nd

^#^ Non-enveloped viruses—**PV1; FCV; AdV5**. Enveloped virus—**IAV**. * SD—standard deviation. Results are means from three independent experiments. **∆Lgs** are calculated as the difference with _d_H_2_O-loaded PNIPAm cryogel. nd—not done.

**Table 2:** Virucidal activity of 65% liquid formic acid (FA) mixed at a ratio of 1:1 with various naked and enveloped RNA and DNA viruses in a suspension test.

Treatment +Viral Model	Final FA Concentration # %	Viral Titers lg CCID_50_/0.1 mL ± SD and Δlg *					
		5 min	∆Lg	15 min	∆Lg	30 min	∆Lg
65% FA + **PV1**	32.5	0.94 ± 1.36	4.11	0.28 ± 0.25	5.0	0.00	5.11
Mock (DMEM) + **PV1**	0	5.05 ± 0.42	-	5.28 ± 0.25	-	5.11 ± 0.71	-
65% FA + **FCV**	32.5	1.19 ± 0.17	3.92	0.5 ± 0.17	4.56	0.00	5.28
Mock (DMEM) + **FCV**	0	5.11 ± 0.54	-	5.05 ± 0.42	-	5.28 ± 0.25	-
65% FA + **AdV5**	32.5	3.19 ± 0.17	0.92	2.05 ± 0.42	1.94	0.00	4.15
Mock (DMEM) + **AdV5**	0	4.11 ± 0.54	-	4.0 ± 0.34	-	4.15 ± 0.25	-
65% FA + IAV	32.5	0.11 ± 0.19	4.78	0.00	5.11	0.00	5.0
Mock (DMEM) + **IAV**	0	4.89 ± 0.51	**-**	5.11 ± 0.71	**-**	5.0 ± 0.34	**-**

Statistical Analysis: Values are expressed as titers (CCID_50_/0.1 mL) determined for each time point. Mean of three experiments ±SD. * SD—standard deviation. **∆lg**—difference between 65% FA and mock (DMEM) control. # **pH** value determined for 65% liquid FA was estimated as 1.0, and for 32.5% liquid FA was estimated between 1.5 and 2.0.

## Data Availability

The original contributions presented in this study are included in the article. Further inquiries can be directed to the corresponding author.
